# Keep Cool

**Published:** 1873-07

**Authors:** 


### The Bistoury.

ELMIRA, N. Y., JULY, 1873.

The BISTOURY is published Quarterly, upon the 1st of April, July, October and January, at Fifty Cents a year, in advance.

### KEEP COOL.

In view of the thermometer registering somewhere in the nineties, this command may be considered ironical. But we are not given to irony, particularly in hot weather,—for we have observed it to be anything but cool employment, no matter what the Farenheit condition of one's surroundings.

To keep cool, it is very essential that one should *be* cool,—that is, in the manner of transacting business, which should be dispatched as far as possible, in the early morning, so that the middle of the day can be given to rest or the enjoyments of the open air and shade. Eat but two meals a day, and let their composition be such as not to increase the temperature of the body. Meats, particularly those of a fatty nature, should be eschewed. Nature furnishes a timely and abundant form of food at this season of the year, entirely wholesome and proper for the requirements of the body. Berries and other fruits, vegetables, bread and milk are the articles offered in abundance and are suited to the occasion. Their consumption will not only conduce to keeping cool, but will be a safeguard against sickness.

Fat people are apt to *lean* upon their avoirdupois as an immunity against disease. This statement may appear paradoxical, but it isn't. Such people should "go slow," (fortunately they cannot do otherwise,—a wise provision of nature, you see), and should do nothing in a hurry. To be fat is to be hot, therefore, nothing should be added to the flame, in the form of alcoholic drinks, to increase this heat. (This is intended as a slight admonition to young ladies who are in the habit of offering wines to their flames; it is an extremely hazardous proceeding for those balancing two hundred, or thereabout). Fat people, to keep cool then, should not partake of wines, liquors, meats and sweet-meats. These articles are rich in carbon, and manufacture heat for the body. Of course, we speak from experience, how could it be otherwise, when our intimate friends photograph us as weighing over two hundred, and bald-headed, a condition certainly calculated to make any one sweat, particularly when engaged upon an article of this character, while the printer is waiting, just outside your private office door, for "copy."

TO KEEP COOL IS TO BE COOL IN ALL CONDITIONS OF BODY AND MIND. Perhaps we penciled that sentence before, or something akin to it,—likely, but we reiterate and reproclaim it, because it's a good thing to say, and we doubt not will look well in type. (If the printer will just place that sentence in small caps, we think it will improve its appearance; upon reflection we are sure it will). One should avoid controversy—like the "Sunday Question," for instance—perplexity, violent exercise, much eating and drinking, or any sort of employment apt to increase one's circulation,—either of blood or money. Of course, such advice to laboring people, or those who are compelled to work, would be idle. But it is for the idle we are writing, or those who can afford to be in that enviable condition, if they will, and whose great desire, for the present moment is, to keep cool, even if they pay roundly for the luxury.

To creep into a refrigerator would be a quick way of solving the problem;—a trip in search of the *Polaris* would be another, but both would be *isolated* instances of emotional insanity, the contemplation of which is calculated to make one hot-headed, therefore uncomfortable.

To go to any of the fashionable watering places, and mimic the style, or strive to outdo, that of your neighbor's would be equally ridiculous. Better remain at home, air your dwelling at early dawn, then close the blinds, darken your rooms and "lay off" in the middle of the day. Otherwise to keep cool, *be* cool.

